# 2-Hy­droxy-11-methyl-16-[(*E*)-4-methyl­benzyl­idene]-13-(4-methyl­phen­yl)-1,11-diaza­penta­cyclo­[12.3.1.0^2,10^.0^3,8^.0^10,14^]octa­deca-3(8),4,6-triene-9,15-dione

**DOI:** 10.1107/S1600536810033064

**Published:** 2010-08-21

**Authors:** Raju Suresh Kumar, Hasnah Osman, Mohamed Ashraf Ali, Chin Sing Yeap, Hoong-Kun Fun

**Affiliations:** a School of Chemical Sciences, Universiti Sains Malaysia, 11800 USM, Penang, Malaysia; b School of Physical Sciences, Universiti Sains Malaysia, 11800 USM, Penang, Malaysia; c X-ray Crystallography Unit, School of Physics, Universiti Sains Malaysia, 11800 USM, Penang, Malaysia

## Abstract

In the title compound, C_32_H_30_N_2_O_3_, the piperidin-4-one and the two fused pyrrolidine rings adopt envelope conformations. The two methyl­phenyl rings are oriented at dihedral angle of 20.36 (7) and 56.24 (7)°, respectively, with respect to the indanone ring system. In the crystal structure, inter­molecular O—H⋯N and C—H⋯O hydrogen bonds link the mol­ecules into chains propagating along [001]. Weak C—H⋯π inter­actions are also observed.

## Related literature

For general background and the biological activity of pyrrolidine compounds, see: Mitchell & Teh (2005 ▶); Okazaki *et al.* (2004 ▶); Enyedy *et al.* (2001 ▶); Yee *et al.* (1998 ▶); Saravanan & Corey (2003 ▶); Crane & Corey (2001 ▶); Xi *et al.* (2004 ▶); Kagan (1975 ▶). For the synthesis, see: Kumar *et al.* (2010*a* ▶,*b* ▶). For ring conformations, see Cremer & Pople (1975 ▶). For the stability of the temperature controller used in the data collection, see: Cosier & Glazer (1986 ▶).
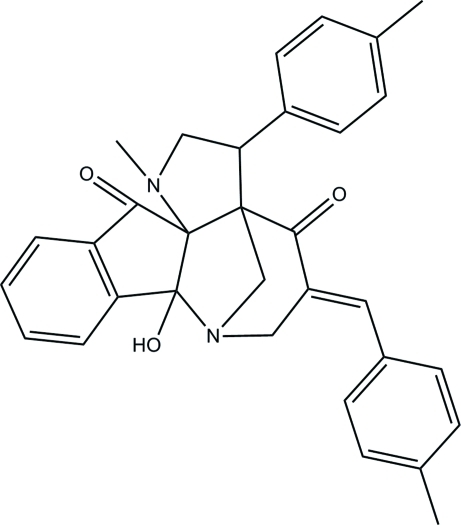


## Experimental

### 

#### Crystal data

C_32_H_30_N_2_O_3_
*M* *_r_* = 490.58
Triclinic, 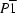

*a* = 9.3252 (6) Å
*b* = 12.1781 (8) Å
*c* = 12.9821 (8) Å
α = 67.480 (2)°
β = 86.093 (2)°
γ = 69.475 (2)°
*V* = 1271.68 (14) Å^3^
*Z* = 2
Cu *K*α radiation
μ = 0.65 mm^−1^
*T* = 100 K
0.56 × 0.29 × 0.25 mm

#### Data collection

Bruker APEXII DUO CCD area-detector diffractometer
Absorption correction: multi-scan (*SADABS*; Bruker, 2009 ▶) *T*_min_ = 0.713, *T*_max_ = 0.851
13699 measured reflections
4121 independent reflections
4004 reflections with *I* > 2σ(*I*)
*R*_int_ = 0.024

#### Refinement

*R*[*F* ^2^ > 2σ(*F* ^2^)] = 0.044
*wR*(*F* ^2^) = 0.134
*S* = 1.21
4121 reflections
342 parameters
H atoms treated by a mixture of independent and constrained refinement
Δρ_max_ = 0.32 e Å^−3^
Δρ_min_ = −0.40 e Å^−3^

### 

Data collection: *APEX2* (Bruker, 2009 ▶); cell refinement: *SAINT* (Bruker, 2009 ▶); data reduction: *SAINT*; program(s) used to solve structure: *SHELXTL* (Sheldrick, 2008 ▶); program(s) used to refine structure: *SHELXTL*; molecular graphics: *SHELXTL*; software used to prepare material for publication: *SHELXTL* and *PLATON* (Spek, 2009 ▶).

## Supplementary Material

Crystal structure: contains datablocks global, I. DOI: 10.1107/S1600536810033064/ci5154sup1.cif

Structure factors: contains datablocks I. DOI: 10.1107/S1600536810033064/ci5154Isup2.hkl

Additional supplementary materials:  crystallographic information; 3D view; checkCIF report

## Figures and Tables

**Table 1:** Hydrogen-bond geometry (Å, °) *Cg*1 is the centroid of the C13–C18 ring.

*D*—H⋯*A*	*D*—H	H⋯*A*	*D*⋯*A*	*D*—H⋯*A*
O2—H1*O*2⋯N2^i^	0.85 (2)	2.00 (2)	2.794 (2)	156 (2)
C5—H5*A*⋯O3^ii^	0.93	2.48	3.206 (2)	135
C11—H11*B*⋯O2^i^	0.97	2.36	3.258 (2)	154
C13—H13*A*⋯O2^i^	0.93	2.40	3.291 (2)	161
C30—H30*C*⋯*Cg*1^iii^	0.96	2.83	3.563 (3)	134

Symmetry codes: (i) 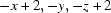; (ii) 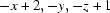; (iii) 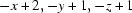.

**Fig. 1.**
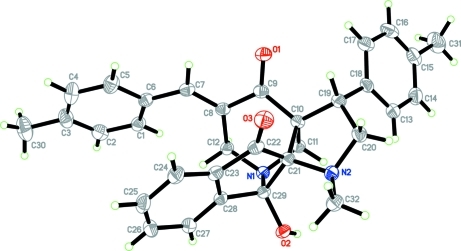
The molecular structure of the title compound, showing 50% probability displacment ellipsoids for non-H atoms and atom labels.

**Fig. 2.**
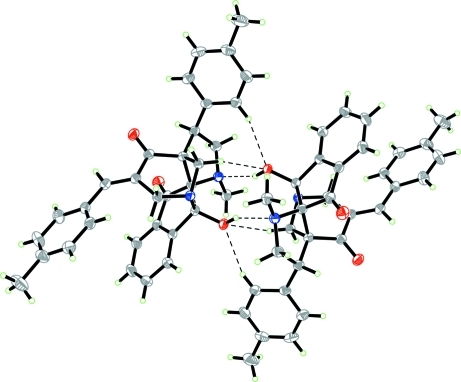
A view of a centrosymmtric dimer in the title compound. Displacement ellipsoids are drawn at the 50% probability level.

**Fig. 3.**
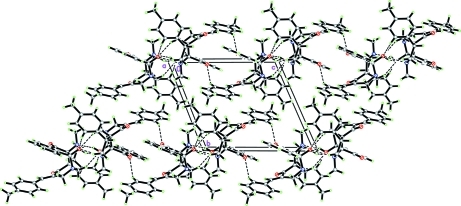
The crystal packing of title compound, viewed down the a axis, showing the dimers being linked into chains along the [001] direction. Intermolecular hydrogen bonds are shown as dashed lines.

## supplementary crystallographic information

### Comment

Substituted pyrrolidine derivatives are widespread structural features of natural and designed biologically active molecules (Mitchell & Teh, 2005; Okazaki *et al.*, 2004; Enyedy *et al.*, 2001). In addition, these heterocycles can be used for pharmaceutical purposes (Yee *et al.*, 1998; Saravanan & Corey, 2003; Crane & Corey, 2001; Xi *et al.*, 2004) and ligands of transition metal catalysts (Kagan, 1975). Consequently, the efficient preparation of these heterocycles has received significant attention. In view of this importance, the crystal structure determination of the title compound was carried out and the results are presented here.

The molecular structure of the title compound is shown in Fig. 1. The piperidin-4-one ring (N1/C12/C8–C11) adopts a distorted envelope conformation (flap atom C11), with puckering parameters Q = 0.625 (2) Å, θ = 140.1 (2)° and φ = 237.9 (2)° (Cremer & Pople, 1975). The two fused pyrrolidine rings with atom sequences N1/C11/C10/C21/C29 and N2/C20/C19/C10/C21 adopt envelope conformations, with atoms C11 and C21, respectively, as flap atoms. The puckering parameters are Q = 0.454 (2) Å, φ = 38.7 (2)° for the N1/C11/C10/C21/C29 pyrrolidine ring and Q = 0.341 (2) Å, φ = 331.2 (3)° for the N2/C20/C19/C10/C21 pyrrolidine ring. The two benzene rings (C1–C6 and C13–C18) make dihedral angle of 20.36 (7) and 56.24 (7)°, respectively with the mean plane of indan-1-one (C21–C29) ring system. The geometric parameters are consistent to those observed in closely related structures (Kumar *et al.*, 2010**a*,b*).

In the crystal structure, intermolecular O2—H1O2···N2, C11—H11B···O2 and C13—H13A···O2 hydrogen bonds (Table 1) link the molecules into dimers (Fig. 2). The dimers are interconnected into chains propagating along the [001] direction *via* intermolecular C5—H5A···O3 hydrogen bonds (Fig. 3 and Table 1). Weak intermolecular C30—H30C···π interactions (Table 1) involving the C13–C18 benzene ring are also observed.

### Experimental

The title compound was synthesized according to the procedure described by Kumar *et al.* (2010*a*,*b*), and was recrystallized from ethyl acetate to afford pale yellow crystals.

### Refinement

The hydroxyl H atom was located in a difference Fourier map and was refined freely. The remaining H atoms were positioned geometrically [C–H = 0.93–0.97 Å] and refined using a riding model, with *U*_iso_(H) = 1.2 or 1.5*U*_eq_(C). A rotating-group model was applied for methyl groups.

### Crystal data

**Table:**

C_32_H_30_N_2_O_3_	*Z* = 2
*M**_r_* = 490.58	*F*(000) = 520
Triclinic, *P*1	*D*_x_ = 1.281 Mg m^−^^3^
Hall symbol: -P 1	Cu *K*α radiation, λ = 1.54178 Å
*a* = 9.3252 (6) Å	Cell parameters from 9995 reflections
*b* = 12.1781 (8) Å	θ = 5.8–67.8°
*c* = 12.9821 (8) Å	µ = 0.65 mm^−^^1^
α = 67.480 (2)°	*T* = 100 K
β = 86.093 (2)°	Plate, yellow
γ = 69.475 (2)°	0.56 × 0.29 × 0.25 mm
*V* = 1271.68 (14) Å^3^	

### Data collection

**Table:**

Bruker APEXII DUO CCD area-detector diffractometer	4121 independent reflections
Radiation source: fine-focus sealed tube	4004 reflections with *I* > 2σ(*I*)
graphite	*R*_int_ = 0.024
φ and ω scans	θ_max_ = 65.0°, θ_min_ = 6.1°
Absorption correction: multi-scan (*SADABS*; Bruker, 2009)	*h* = −8→10
*T*_min_ = 0.713, *T*_max_ = 0.851	*k* = −14→14
13699 measured reflections	*l* = −15→15

### Refinement

**Table:**

Refinement on *F*^2^	Secondary atom site location: difference Fourier map
Least-squares matrix: full	Hydrogen site location: inferred from neighbouring sites
*R*[*F*^2^ > 2σ(*F*^2^)] = 0.044	H atoms treated by a mixture of independent and constrained refinement
*wR*(*F*^2^) = 0.134	*w* = 1/[σ^2^(*F*_o_^2^) + (0.0743*P*)^2^ + 0.3694*P*] where *P* = (*F*_o_^2^ + 2*F*_c_^2^)/3
*S* = 1.21	(Δ/σ)_max_ = 0.001
4121 reflections	Δρ_max_ = 0.32 e Å^−^^3^
342 parameters	Δρ_min_ = −0.40 e Å^−^^3^
0 restraints	Extinction correction: *SHELXTL* (Sheldrick, 2008), Fc^*^=kFc[1+0.001xFc^2^λ^3^/sin(2θ)]^-1/4^
Primary atom site location: structure-invariant direct methods	Extinction coefficient: 0.069 (4)

### Special details

**Table:**

Experimental. The crystal was placed in the cold stream of an Oxford Cryosystems Cobra open-flow nitrogen cryostat (Cosier & Glazer, 1986) operating at 100.0 (1) K.
Geometry. All e.s.d.'s (except the e.s.d. in the dihedral angle between two l.s. planes) are estimated using the full covariance matrix. The cell e.s.d.'s are taken into account individually in the estimation of e.s.d.'s in distances, angles and torsion angles; correlations between e.s.d.'s in cell parameters are only used when they are defined by crystal symmetry. An approximate (isotropic) treatment of cell e.s.d.'s is used for estimating e.s.d.'s involving l.s. planes.
Refinement. Refinement of *F*^2^ against ALL reflections. The weighted *R*-factor *wR* and goodness of fit *S* are based on *F*^2^, conventional *R*-factors *R* are based on *F*, with *F* set to zero for negative *F*^2^. The threshold expression of *F*^2^ > σ(*F*^2^) is used only for calculating *R*-factors(gt) *etc*. and is not relevant to the choice of reflections for refinement. *R*-factors based on *F*^2^ are statistically about twice as large as those based on *F*, and *R*- factors based on ALL data will be even larger.

### Fractional atomic coordinates and isotropic or equivalent isotropic displacement parameters (Å^2^)

**Table:**

	*x*	*y*	*z*	*U*_iso_*/*U*_eq_	
O1	0.69853 (13)	0.26369 (11)	0.55957 (9)	0.0290 (3)	
O2	1.14244 (12)	−0.01927 (10)	0.92987 (8)	0.0219 (3)	
O3	0.90572 (13)	−0.06206 (11)	0.65669 (9)	0.0296 (3)	
N1	1.02023 (14)	0.19002 (11)	0.79859 (10)	0.0195 (3)	
N2	0.87997 (14)	−0.05467 (11)	0.88897 (10)	0.0213 (3)	
C1	1.18437 (18)	0.39376 (14)	0.46558 (13)	0.0255 (4)	
H1A	1.1585	0.4127	0.5288	0.031*	
C2	1.30607 (19)	0.42114 (15)	0.40771 (14)	0.0306 (4)	
H2A	1.3609	0.4577	0.4332	0.037*	
C3	1.3481 (2)	0.39515 (16)	0.31222 (15)	0.0344 (4)	
C4	1.2616 (2)	0.34286 (17)	0.27535 (15)	0.0374 (4)	
H4A	1.2859	0.3264	0.2108	0.045*	
C5	1.1405 (2)	0.31491 (16)	0.33248 (14)	0.0314 (4)	
H5A	1.0848	0.2800	0.3056	0.038*	
C6	1.09964 (18)	0.33786 (14)	0.42997 (12)	0.0238 (4)	
C7	0.97216 (17)	0.30238 (13)	0.48655 (12)	0.0230 (4)	
H7A	0.9031	0.2979	0.4412	0.028*	
C8	0.93923 (17)	0.27523 (13)	0.59393 (12)	0.0207 (3)	
C9	0.80311 (17)	0.23458 (13)	0.62724 (12)	0.0215 (3)	
C10	0.80285 (17)	0.15433 (13)	0.75147 (12)	0.0202 (3)	
C11	0.85546 (16)	0.21946 (13)	0.81648 (12)	0.0202 (3)	
H11A	0.8003	0.3104	0.7865	0.024*	
H11B	0.8398	0.1847	0.8954	0.024*	
C12	1.03611 (17)	0.27348 (14)	0.68483 (12)	0.0208 (3)	
H12A	1.1432	0.2463	0.6690	0.025*	
H12B	1.0074	0.3593	0.6820	0.025*	
C13	0.52758 (18)	0.21075 (15)	0.92567 (13)	0.0279 (4)	
H13A	0.6058	0.1488	0.9792	0.033*	
C14	0.4105 (2)	0.29883 (16)	0.95461 (14)	0.0324 (4)	
H14A	0.4123	0.2955	1.0273	0.039*	
C15	0.29066 (19)	0.39205 (16)	0.87786 (15)	0.0333 (4)	
C16	0.29137 (19)	0.39398 (16)	0.77021 (15)	0.0333 (4)	
H16A	0.2117	0.4547	0.7174	0.040*	
C17	0.40876 (18)	0.30691 (15)	0.74029 (14)	0.0282 (4)	
H17A	0.4070	0.3106	0.6675	0.034*	
C18	0.52947 (17)	0.21387 (14)	0.81723 (13)	0.0233 (4)	
C19	0.65657 (17)	0.12088 (14)	0.78116 (12)	0.0227 (4)	
H19A	0.6158	0.1213	0.7133	0.027*	
C20	0.71422 (18)	−0.01858 (14)	0.86592 (13)	0.0250 (4)	
H20A	0.6621	−0.0261	0.9342	0.030*	
H20B	0.6947	−0.0732	0.8347	0.030*	
C21	0.93759 (16)	0.02464 (13)	0.79105 (12)	0.0199 (3)	
C22	0.98737 (18)	−0.02951 (13)	0.69987 (12)	0.0223 (3)	
C23	1.14573 (18)	−0.03556 (14)	0.67710 (12)	0.0226 (3)	
C24	1.23947 (19)	−0.08211 (15)	0.60366 (13)	0.0271 (4)	
H24A	1.2029	−0.1121	0.5589	0.033*	
C25	1.3875 (2)	−0.08246 (16)	0.59918 (13)	0.0308 (4)	
H25A	1.4519	−0.1129	0.5507	0.037*	
C26	1.44221 (19)	−0.03750 (16)	0.66696 (13)	0.0297 (4)	
H26A	1.5428	−0.0391	0.6632	0.036*	
C27	1.34872 (18)	0.00941 (15)	0.73970 (12)	0.0249 (4)	
H27A	1.3855	0.0392	0.7846	0.030*	
C28	1.19917 (17)	0.01076 (13)	0.74373 (11)	0.0204 (3)	
C29	1.07917 (17)	0.05268 (13)	0.81917 (11)	0.0193 (3)	
C30	1.4823 (3)	0.4233 (2)	0.25134 (19)	0.0510 (6)	
H30A	1.5176	0.3737	0.2062	0.077*	
H30B	1.5639	0.4021	0.3046	0.077*	
H30C	1.4510	0.5119	0.2043	0.077*	
C31	0.1659 (2)	0.4899 (2)	0.9091 (2)	0.0500 (5)	
H31A	0.0674	0.4950	0.8860	0.075*	
H31B	0.1772	0.5712	0.8725	0.075*	
H31C	0.1737	0.4660	0.9886	0.075*	
C32	0.96162 (18)	−0.19190 (14)	0.92734 (13)	0.0253 (4)	
H32A	0.9288	−0.2336	0.9988	0.038*	
H32B	1.0701	−0.2098	0.9341	0.038*	
H32C	0.9395	−0.2223	0.8743	0.038*	
H1O2	1.115 (3)	0.022 (2)	0.972 (2)	0.053 (7)*	

### Atomic displacement parameters (Å^2^)

**Table:**

	*U*^11^	*U*^22^	*U*^33^	*U*^12^	*U*^13^	*U*^23^
O1	0.0255 (6)	0.0357 (6)	0.0235 (6)	−0.0126 (5)	−0.0034 (4)	−0.0066 (5)
O2	0.0243 (6)	0.0219 (5)	0.0167 (5)	−0.0056 (4)	−0.0005 (4)	−0.0066 (4)
O3	0.0326 (7)	0.0326 (6)	0.0303 (6)	−0.0145 (5)	0.0000 (5)	−0.0161 (5)
N1	0.0195 (7)	0.0199 (6)	0.0185 (6)	−0.0072 (5)	0.0011 (5)	−0.0064 (5)
N2	0.0214 (7)	0.0199 (6)	0.0215 (6)	−0.0079 (5)	0.0020 (5)	−0.0062 (5)
C1	0.0284 (9)	0.0224 (8)	0.0224 (8)	−0.0100 (6)	0.0013 (6)	−0.0043 (6)
C2	0.0295 (9)	0.0241 (8)	0.0351 (9)	−0.0120 (7)	0.0003 (7)	−0.0054 (7)
C3	0.0312 (10)	0.0253 (8)	0.0404 (10)	−0.0094 (7)	0.0115 (7)	−0.0078 (7)
C4	0.0472 (11)	0.0354 (9)	0.0345 (9)	−0.0191 (8)	0.0192 (8)	−0.0171 (8)
C5	0.0397 (10)	0.0305 (9)	0.0287 (9)	−0.0176 (7)	0.0070 (7)	−0.0123 (7)
C6	0.0260 (8)	0.0199 (7)	0.0206 (7)	−0.0073 (6)	0.0005 (6)	−0.0033 (6)
C7	0.0247 (8)	0.0205 (7)	0.0224 (8)	−0.0084 (6)	−0.0025 (6)	−0.0057 (6)
C8	0.0213 (8)	0.0174 (7)	0.0211 (7)	−0.0061 (6)	−0.0017 (6)	−0.0051 (6)
C9	0.0214 (8)	0.0198 (7)	0.0224 (8)	−0.0052 (6)	−0.0009 (6)	−0.0088 (6)
C10	0.0181 (8)	0.0204 (7)	0.0217 (7)	−0.0065 (6)	0.0001 (6)	−0.0077 (6)
C11	0.0194 (8)	0.0197 (7)	0.0207 (7)	−0.0061 (6)	0.0013 (5)	−0.0075 (6)
C12	0.0216 (8)	0.0206 (7)	0.0197 (7)	−0.0092 (6)	0.0012 (6)	−0.0054 (6)
C13	0.0215 (8)	0.0297 (8)	0.0264 (8)	−0.0074 (6)	0.0029 (6)	−0.0061 (6)
C14	0.0293 (9)	0.0351 (9)	0.0309 (9)	−0.0113 (7)	0.0099 (7)	−0.0119 (7)
C15	0.0224 (9)	0.0306 (9)	0.0456 (10)	−0.0099 (7)	0.0082 (7)	−0.0137 (8)
C16	0.0209 (9)	0.0275 (8)	0.0448 (10)	−0.0048 (6)	−0.0060 (7)	−0.0091 (7)
C17	0.0244 (8)	0.0277 (8)	0.0314 (8)	−0.0098 (6)	−0.0029 (6)	−0.0090 (7)
C18	0.0177 (8)	0.0236 (8)	0.0285 (8)	−0.0104 (6)	0.0022 (6)	−0.0070 (6)
C19	0.0209 (8)	0.0245 (8)	0.0229 (7)	−0.0094 (6)	−0.0003 (6)	−0.0075 (6)
C20	0.0226 (8)	0.0244 (8)	0.0277 (8)	−0.0103 (6)	0.0025 (6)	−0.0080 (6)
C21	0.0197 (8)	0.0193 (7)	0.0197 (7)	−0.0071 (6)	0.0007 (6)	−0.0059 (6)
C22	0.0262 (8)	0.0185 (7)	0.0205 (7)	−0.0073 (6)	−0.0017 (6)	−0.0058 (6)
C23	0.0258 (8)	0.0199 (7)	0.0195 (7)	−0.0064 (6)	0.0003 (6)	−0.0060 (6)
C24	0.0329 (9)	0.0269 (8)	0.0222 (8)	−0.0095 (7)	0.0035 (6)	−0.0112 (6)
C25	0.0340 (9)	0.0314 (9)	0.0253 (8)	−0.0090 (7)	0.0109 (7)	−0.0128 (7)
C26	0.0231 (9)	0.0330 (9)	0.0310 (9)	−0.0094 (7)	0.0072 (6)	−0.0114 (7)
C27	0.0237 (8)	0.0274 (8)	0.0233 (8)	−0.0093 (6)	0.0023 (6)	−0.0095 (6)
C28	0.0213 (8)	0.0189 (7)	0.0173 (7)	−0.0056 (6)	0.0007 (5)	−0.0043 (6)
C29	0.0204 (8)	0.0197 (7)	0.0169 (7)	−0.0070 (6)	−0.0001 (5)	−0.0060 (6)
C30	0.0444 (12)	0.0444 (11)	0.0659 (14)	−0.0230 (9)	0.0275 (10)	−0.0200 (10)
C31	0.0335 (11)	0.0453 (11)	0.0627 (14)	−0.0031 (9)	0.0119 (9)	−0.0231 (10)
C32	0.0290 (9)	0.0197 (8)	0.0251 (8)	−0.0080 (6)	0.0013 (6)	−0.0067 (6)

### Geometric parameters (Å, °)

**Table:**

O1—C9	1.2183 (18)	C14—C15	1.388 (2)
O2—C29	1.4033 (17)	C14—H14A	0.93
O2—H1O2	0.85 (3)	C15—C16	1.388 (3)
O3—C22	1.2211 (18)	C15—C31	1.507 (2)
N1—C12	1.4703 (18)	C16—C17	1.385 (2)
N1—C11	1.4738 (19)	C16—H16A	0.93
N1—C29	1.4845 (18)	C17—C18	1.393 (2)
N2—C32	1.4637 (19)	C17—H17A	0.93
N2—C20	1.469 (2)	C18—C19	1.515 (2)
N2—C21	1.4723 (18)	C19—C20	1.547 (2)
C1—C2	1.386 (2)	C19—H19A	0.98
C1—C6	1.403 (2)	C20—H20A	0.97
C1—H1A	0.93	C20—H20B	0.97
C2—C3	1.392 (3)	C21—C22	1.536 (2)
C2—H2A	0.93	C21—C29	1.572 (2)
C3—C4	1.390 (3)	C22—C23	1.468 (2)
C3—C30	1.505 (2)	C23—C28	1.394 (2)
C4—C5	1.380 (2)	C23—C24	1.396 (2)
C4—H4A	0.93	C24—C25	1.376 (2)
C5—C6	1.399 (2)	C24—H24A	0.93
C5—H5A	0.93	C25—C26	1.400 (2)
C6—C7	1.462 (2)	C25—H25A	0.93
C7—C8	1.344 (2)	C26—C27	1.389 (2)
C7—H7A	0.93	C26—H26A	0.93
C8—C9	1.498 (2)	C27—C28	1.387 (2)
C8—C12	1.524 (2)	C27—H27A	0.93
C9—C10	1.533 (2)	C28—C29	1.518 (2)
C10—C19	1.540 (2)	C30—H30A	0.96
C10—C21	1.5530 (19)	C30—H30B	0.96
C10—C11	1.5569 (19)	C30—H30C	0.96
C11—H11A	0.97	C31—H31A	0.96
C11—H11B	0.97	C31—H31B	0.96
C12—H12A	0.97	C31—H31C	0.96
C12—H12B	0.97	C32—H32A	0.96
C13—C14	1.384 (2)	C32—H32B	0.96
C13—C18	1.393 (2)	C32—H32C	0.96
C13—H13A	0.93		
			
C29—O2—H1O2	112.9 (16)	C13—C18—C17	117.73 (15)
C12—N1—C11	108.58 (11)	C13—C18—C19	122.55 (14)
C12—N1—C29	114.54 (11)	C17—C18—C19	119.72 (14)
C11—N1—C29	104.38 (11)	C18—C19—C10	114.91 (12)
C32—N2—C20	112.70 (12)	C18—C19—C20	115.93 (13)
C32—N2—C21	116.01 (12)	C10—C19—C20	104.77 (11)
C20—N2—C21	107.99 (11)	C18—C19—H19A	106.9
C2—C1—C6	120.88 (15)	C10—C19—H19A	106.9
C2—C1—H1A	119.6	C20—C19—H19A	106.9
C6—C1—H1A	119.6	N2—C20—C19	106.63 (12)
C1—C2—C3	121.48 (16)	N2—C20—H20A	110.4
C1—C2—H2A	119.3	C19—C20—H20A	110.4
C3—C2—H2A	119.3	N2—C20—H20B	110.4
C4—C3—C2	117.59 (16)	C19—C20—H20B	110.4
C4—C3—C30	121.49 (17)	H20A—C20—H20B	108.6
C2—C3—C30	120.92 (18)	N2—C21—C22	114.83 (11)
C5—C4—C3	121.40 (16)	N2—C21—C10	102.80 (11)
C5—C4—H4A	119.3	C22—C21—C10	114.00 (11)
C3—C4—H4A	119.3	N2—C21—C29	114.35 (11)
C4—C5—C6	121.44 (16)	C22—C21—C29	105.28 (11)
C4—C5—H5A	119.3	C10—C21—C29	105.45 (11)
C6—C5—H5A	119.3	O3—C22—C23	127.68 (14)
C5—C6—C1	117.17 (14)	O3—C22—C21	123.96 (14)
C5—C6—C7	117.71 (14)	C23—C22—C21	108.36 (12)
C1—C6—C7	125.12 (14)	C28—C23—C24	121.26 (15)
C8—C7—C6	130.04 (14)	C28—C23—C22	110.20 (13)
C8—C7—H7A	115.0	C24—C23—C22	128.53 (14)
C6—C7—H7A	115.0	C25—C24—C23	118.28 (15)
C7—C8—C9	116.81 (13)	C25—C24—H24A	120.9
C7—C8—C12	124.92 (14)	C23—C24—H24A	120.9
C9—C8—C12	118.11 (12)	C24—C25—C26	120.64 (15)
O1—C9—C8	121.97 (13)	C24—C25—H25A	119.7
O1—C9—C10	121.88 (14)	C26—C25—H25A	119.7
C8—C9—C10	116.16 (12)	C27—C26—C25	121.11 (15)
C9—C10—C19	113.59 (12)	C27—C26—H26A	119.4
C9—C10—C21	112.47 (12)	C25—C26—H26A	119.4
C19—C10—C21	105.14 (11)	C28—C27—C26	118.36 (14)
C9—C10—C11	105.65 (11)	C28—C27—H27A	120.8
C19—C10—C11	119.93 (12)	C26—C27—H27A	120.8
C21—C10—C11	99.28 (11)	C27—C28—C23	120.35 (14)
N1—C11—C10	103.44 (11)	C27—C28—C29	127.57 (13)
N1—C11—H11A	111.1	C23—C28—C29	112.01 (13)
C10—C11—H11A	111.1	O2—C29—N1	110.03 (11)
N1—C11—H11B	111.1	O2—C29—C28	107.24 (11)
C10—C11—H11B	111.1	N1—C29—C28	116.66 (12)
H11A—C11—H11B	109.0	O2—C29—C21	113.48 (11)
N1—C12—C8	114.17 (12)	N1—C29—C21	105.34 (11)
N1—C12—H12A	108.7	C28—C29—C21	104.15 (11)
C8—C12—H12A	108.7	C3—C30—H30A	109.5
N1—C12—H12B	108.7	C3—C30—H30B	109.5
C8—C12—H12B	108.7	H30A—C30—H30B	109.5
H12A—C12—H12B	107.6	C3—C30—H30C	109.5
C14—C13—C18	120.67 (15)	H30A—C30—H30C	109.5
C14—C13—H13A	119.7	H30B—C30—H30C	109.5
C18—C13—H13A	119.7	C15—C31—H31A	109.5
C13—C14—C15	121.66 (16)	C15—C31—H31B	109.5
C13—C14—H14A	119.2	H31A—C31—H31B	109.5
C15—C14—H14A	119.2	C15—C31—H31C	109.5
C14—C15—C16	117.66 (15)	H31A—C31—H31C	109.5
C14—C15—C31	121.37 (17)	H31B—C31—H31C	109.5
C16—C15—C31	120.96 (17)	N2—C32—H32A	109.5
C17—C16—C15	121.05 (15)	N2—C32—H32B	109.5
C17—C16—H16A	119.5	H32A—C32—H32B	109.5
C15—C16—H16A	119.5	N2—C32—H32C	109.5
C16—C17—C18	121.23 (16)	H32A—C32—H32C	109.5
C16—C17—H17A	119.4	H32B—C32—H32C	109.5
C18—C17—H17A	119.4		
			
C6—C1—C2—C3	0.4 (2)	C32—N2—C21—C22	39.35 (17)
C1—C2—C3—C4	1.3 (2)	C20—N2—C21—C22	−88.23 (14)
C1—C2—C3—C30	−178.91 (16)	C32—N2—C21—C10	163.73 (12)
C2—C3—C4—C5	−1.4 (3)	C20—N2—C21—C10	36.15 (14)
C30—C3—C4—C5	178.74 (17)	C32—N2—C21—C29	−82.52 (15)
C3—C4—C5—C6	−0.1 (3)	C20—N2—C21—C29	149.89 (12)
C4—C5—C6—C1	1.7 (2)	C9—C10—C21—N2	−155.97 (11)
C4—C5—C6—C7	−178.52 (15)	C19—C10—C21—N2	−31.88 (13)
C2—C1—C6—C5	−1.9 (2)	C11—C10—C21—N2	92.73 (12)
C2—C1—C6—C7	178.37 (14)	C9—C10—C21—C22	−31.04 (17)
C5—C6—C7—C8	156.33 (16)	C19—C10—C21—C22	93.04 (14)
C1—C6—C7—C8	−23.9 (3)	C11—C10—C21—C22	−142.35 (12)
C6—C7—C8—C9	−176.56 (14)	C9—C10—C21—C29	83.93 (13)
C6—C7—C8—C12	−1.3 (3)	C19—C10—C21—C29	−151.98 (11)
C7—C8—C9—O1	−24.1 (2)	C11—C10—C21—C29	−27.37 (13)
C12—C8—C9—O1	160.29 (14)	N2—C21—C22—O3	53.63 (19)
C7—C8—C9—C10	156.00 (13)	C10—C21—C22—O3	−64.61 (19)
C12—C8—C9—C10	−19.64 (18)	C29—C21—C22—O3	−179.69 (13)
O1—C9—C10—C19	−2.6 (2)	N2—C21—C22—C23	−125.69 (13)
C8—C9—C10—C19	177.34 (12)	C10—C21—C22—C23	116.07 (13)
O1—C9—C10—C21	116.68 (15)	C29—C21—C22—C23	0.98 (14)
C8—C9—C10—C21	−63.39 (16)	O3—C22—C23—C28	179.97 (14)
O1—C9—C10—C11	−136.04 (14)	C21—C22—C23—C28	−0.75 (16)
C8—C9—C10—C11	43.90 (15)	O3—C22—C23—C24	−1.6 (3)
C12—N1—C11—C10	76.80 (13)	C21—C22—C23—C24	177.64 (14)
C29—N1—C11—C10	−45.79 (13)	C28—C23—C24—C25	0.6 (2)
C9—C10—C11—N1	−71.79 (13)	C22—C23—C24—C25	−177.60 (15)
C19—C10—C11—N1	158.37 (12)	C23—C24—C25—C26	0.2 (2)
C21—C10—C11—N1	44.82 (13)	C24—C25—C26—C27	−0.5 (3)
C11—N1—C12—C8	−50.37 (15)	C25—C26—C27—C28	0.0 (2)
C29—N1—C12—C8	65.83 (16)	C26—C27—C28—C23	0.8 (2)
C7—C8—C12—N1	−154.02 (14)	C26—C27—C28—C29	177.55 (14)
C9—C8—C12—N1	21.23 (18)	C24—C23—C28—C27	−1.1 (2)
C18—C13—C14—C15	0.7 (3)	C22—C23—C28—C27	177.39 (13)
C13—C14—C15—C16	0.3 (3)	C24—C23—C28—C29	−178.35 (13)
C13—C14—C15—C31	−178.27 (17)	C22—C23—C28—C29	0.17 (17)
C14—C15—C16—C17	−0.8 (2)	C12—N1—C29—O2	145.71 (12)
C31—C15—C16—C17	177.68 (17)	C11—N1—C29—O2	−95.69 (12)
C15—C16—C17—C18	0.5 (3)	C12—N1—C29—C28	23.31 (17)
C14—C13—C18—C17	−1.0 (2)	C11—N1—C29—C28	141.90 (12)
C14—C13—C18—C19	178.92 (15)	C12—N1—C29—C21	−91.62 (13)
C16—C17—C18—C13	0.5 (2)	C11—N1—C29—C21	26.98 (13)
C16—C17—C18—C19	−179.51 (14)	C27—C28—C29—O2	−55.96 (19)
C13—C18—C19—C10	−81.59 (18)	C23—C28—C29—O2	121.01 (13)
C17—C18—C19—C10	98.37 (16)	C27—C28—C29—N1	67.89 (19)
C13—C18—C19—C20	41.0 (2)	C23—C28—C29—N1	−115.14 (14)
C17—C18—C19—C20	−139.06 (15)	C27—C28—C29—C21	−176.52 (14)
C9—C10—C19—C18	−91.75 (15)	C23—C28—C29—C21	0.45 (15)
C21—C10—C19—C18	144.87 (12)	N2—C21—C29—O2	9.84 (17)
C11—C10—C19—C18	34.47 (18)	C22—C21—C29—O2	−117.13 (12)
C9—C10—C19—C20	139.85 (12)	C10—C21—C29—O2	122.01 (12)
C21—C10—C19—C20	16.48 (14)	N2—C21—C29—N1	−110.58 (12)
C11—C10—C19—C20	−93.92 (15)	C22—C21—C29—N1	122.45 (11)
C32—N2—C20—C19	−155.70 (12)	C10—C21—C29—N1	1.59 (14)
C21—N2—C20—C19	−26.24 (15)	N2—C21—C29—C28	126.12 (12)
C18—C19—C20—N2	−122.78 (13)	C22—C21—C29—C28	−0.85 (14)
C10—C19—C20—N2	4.99 (15)	C10—C21—C29—C28	−121.71 (12)

### Hydrogen-bond geometry (Å, °)

**Table:**

Cg1 is the centroid of the C13–C18 ring.

**Table:**

*D*—H···*A*	*D*—H	H···*A*	*D*···*A*	*D*—H···*A*
O2—H1O2···N2^i^	0.85 (2)	2.00 (2)	2.794 (2)	156 (2)
C5—H5A···O3^ii^	0.93	2.48	3.206 (2)	135
C11—H11B···O2^i^	0.97	2.36	3.258 (2)	154
C13—H13A···O2^i^	0.93	2.40	3.291 (2)	161
C30—H30C···Cg1^iii^	0.96	2.83	3.563 (3)	134

Symmetry codes: (i) −*x*+2, −*y*, −*z*+2; (ii) −*x*+2, −*y*, −*z*+1; (iii) −*x*+2, −*y*+1, −*z*+1.

## References

[bb1] Bruker (2009). *APEX2*, *SAINT* and *SADABS* Bruker AXS Inc., Madison, Wisconsin, USA.

[bb2] Cosier, J. & Glazer, A. M. (1986). *J. Appl. Cryst.* **19**, 105–107.

[bb3] Crane, S. N. & Corey, E. J. (2001). *Org. Lett.* **3**, 1395–1397. 10.1021/ol015757p 11348243

[bb4] Cremer, D. & Pople, J. A. (1975). *J. Am. Chem. Soc.* **97**, 1354–1358.

[bb5] Enyedy, I. J., Zaman, W. A., Sakamuri, S., Kozikowski, A. P., Johnson, K. M. & Wang, S. (2001). *Bioorg. Med. Chem. Lett.* **11**, 1113–1118. 10.1016/s0960-894x(01)00132-9 11354356

[bb6] Kagan, H. B. (1975). *Asymmetric Synthesis*, Vol. 5, ch. 1, edited by J. D. Morrison. Academic Press: New York.

[bb7] Kumar, R. S., Osman, H., Ali, M. A., Hemamalini, M. & Fun, H.-K. (2010*a*). *Acta Cryst.* E**66**, o1370–o1371. 10.1107/S1600536810017216 PMC2979481 21579453

[bb8] Kumar, R. S., Osman, H., Ali, M. A., Quah, C. K. & Fun, H.-K. (2010*b*). *Acta Cryst.* E**66**, o1540–o1541. 10.1107/S1600536810020271 PMC3006797 21587787

[bb9] Mitchell, R. E. & Teh, K. L. (2005). *Org. Biomol. Chem.* **3**, 3540–3543. 10.1039/b509319h 16172692

[bb10] Okazaki, Y., Ishihara, A., Nishida, T. & Iwamura, H. (2004). *Tetrahedron*, **60**, 4765–4771.

[bb11] Saravanan, P. & Corey, E. J. (2003). *J. Org. Chem.* **68**, 2760–2764. 10.1021/jo0268916 12662049

[bb12] Sheldrick, G. M. (2008). *Acta Cryst.* A**64**, 112–122. 10.1107/S0108767307043930 18156677

[bb13] Spek, A. L. (2009). *Acta Cryst.* D**65**, 148–155. 10.1107/S090744490804362X PMC2631630 19171970

[bb14] Xi, N., Arvedson, S., Eisenberg, S., Han, N., Handley, M., Huang, L., Huang, Q., Kiselyov, A., Liu, Q., Lu, Y., Nunez, G., Osslund, T., Powers, D., Tasker, A. S., Wang, L., Xiang, T., Xu, S., Zhang, J., Zhu, J., Kendall, R. & Dominguez, C. (2004). *Bioorg. Med. Chem. Lett.* **14**, 2905–2909. 10.1016/j.bmcl.2004.03.033 15125957

[bb15] Yee, N. K., Nummy, L. J., Byrne, D. P., Smith, L. L. & Roth, G. P. (1998). *J. Org. Chem.* **63**, 326–330.

